# Pulmonary Arterial Hypertension and Cancer: Unveiling Parallels in Epidemiology, Clinical Pathways, and Therapeutic Strategies

**DOI:** 10.3390/jmahp14010009

**Published:** 2026-02-06

**Authors:** Karim EI-Kersh, Nadine Zawadzki, Catelyn Coyle, Shurui Zhang, Dhruv Dalal, Anna Watzker, Dominik Lautsch, Jason Shafrin

**Affiliations:** 1 College of Medicine, University of Arizona, Phoenix, AZ 85004, USA; 2 Center for Healthcare Economics and Policy, FTI Consulting, Los Angeles, CA 90071, USA; 3 Merck & Co., Inc., Rahway, NJ 07065, USA; 4 Center for Healthcare Economics and Policy, FTI Consulting, Washington, DC 20004, USA

**Keywords:** cancer, disease analog, evidence synthesis, pulmonary arterial hypertension

## Abstract

Pulmonary arterial hypertension (PAH) and cancer share high mortality and complex prognoses. Due to PAH’s rarity, these parallels may be underrecognized by healthcare stakeholders. This study explored similarities between PAH and cancer across epidemiological, clinical, therapeutic, and healthcare resource utilization (HCRU) considerations. A four-step approach was employed: (1) inclusion/exclusion criteria were applied to identify potential PAH cancer analogs; (2) characteristics for comparison were categorized as epidemiologic, clinical, therapeutic landscape, and HCRU; (3) a targeted literature review extracted data on disease characteristics; (4) a similarity ranking was calculated as the absolute difference between each cancer’s and PAH’s characteristics. Fourteen cancers met the inclusion criteria. Well-differentiated thyroid cancer (WDTC) had the highest number (5) of characteristics closest to PAH. WDTC and medullary thyroid cancer were most similar to PAH in epidemiology; gastrointestinal stromal tumor was most similar in clinical and HCRU characteristics, and anaplastic lymphoma kinase-positive (ALK+) non-small-cell lung cancer and renal cell carcinoma were most similar in therapeutic landscape. Although no single cancer fully mirrors PAH, the identification of multiple analogs underscores PAH’s multidimensional complexity and confirms its overlap with oncological conditions. Cancer analogs could serve as a valuable framework for enhancing recognition of PAH’s clinical, therapeutic, and HRCU implications among healthcare stakeholders.

## 1. Introduction

Pulmonary hypertension (PH) is characterized by elevated pressure within the pulmonary artery and is classified into five diagnostic groups. Group 2 PH, due to left heart disease, and Group 3 PH, associated with chronic lung disease, are the two most common forms [1]. While PH overall is not rare, Group 1 PH—also known as pulmonary arterial hypertension (PAH)—is a relatively rare disease [2]. Moreover, PAH represents one of the most challenging subtypes of PH, marked by its progressive nature and devastating impact on patient survival [3,4]. Despite therapeutic advances, PAH maintains a sobering prognosis, with transplant-free survival rates of only 57% at five years and 35% at ten years [5]. The disease’s initial non-specific presentation—including dyspnea, fatigue, and dizziness—often leads to critical delays in diagnosis and treatment initiation, potentially compromising patient outcomes [3,4].

An emerging paradigm in PAH pathobiology draws striking parallels with cancer biology. Both conditions share fundamental molecular mechanisms, including deregulated cellular metabolism, sustained proliferation, resistance to apoptosis, and vascular remodeling [5,6]. Clinically, PAH and cancer also share commonalities in their manifestation, such as high mortality, clinical complexity, a progressive nature, and prognosis varying by disease stage and presence/absence of biomarkers [6,7].

While PH specialists recognize these cancer-like features of PAH, this conceptual framework remains underutilized among general practitioners, payers (i.e., health insurers), and healthcare policymakers. This knowledge gap, exacerbated by PAH’s relative rarity, may impact clinical decision-making and healthcare policy development. Drawing parallels between PAH and more widely recognized diseases—particularly cancers—could enhance the understanding of PAH’s severity and complexity among clinicians and decision-makers who influence treatment access and research funding.

This study aims to identify specific cancer types that mirror PAH across multiple domains: epidemiological, clinical manifestation, therapeutic landscape, and healthcare resource utilization (HCRU) characteristics. By establishing these disease analogs, we aim to create a more accessible framework for understanding PAH’s severity and complexity, potentially catalyzing improved disease recognition, research funding, and patient outcomes.

## 2. Materials and Methods

### 2.1. Study Design and Setting

This study involved an evidence synthesis conducted using a four-step approach: (1) selection of potential cancer analogs for comparison to PAH, (2) determination of disease-level characteristics for comparison, (3) targeted literature review and extraction of recent published data on characteristics, and (4) evaluation of cancer analogs most similar to PAH through numeric comparison of the disease-level characteristics values. The primary outcome of the study was the identification of cancer analogs determined to be most similar to PAH. This study was exempt from institutional review board oversight. Additional methodological details are provided in the Supplemental Materials with supporting literature sources that further justify the analytic approach [8,9,10,11,12,13,14,15,16].

### 2.2. Selection Criteria for Cancer Analogs

Cancer analogs were defined by primary tumor site and presence/absence of prognostic biomarker, if any. Cancer analogs were identified through searching for anti-cancer drug product indications in the Food and Drug Administration (FDA) Online Label Repository and the Orphan Drug Designations and Approvals Repository [17], and were determined eligible for comparison to PAH if they had ≥3 FDA-approved anticancer therapeutics, excluding supportive care, adjuvant, or radiologic treatments, at the time of data collection, with ≥1 approved 2013–2023, and United States (US) annual incidence ≥1000 and ≤95,000. Cancers that are hematological, pediatric-onset, sex-specific, secondary to AIDS, or secondary to cancer treatment were excluded (see Table S1 in the Supplemental Materials for details).

### 2.3. Disease-Level Characteristics for Comparison

The similarity of potential cancer analogs to PAH was determined based on a simple numerical comparison of disease-level characteristics across epidemiological, clinical, therapeutic landscape, and HCRU dimensions. Data was collected via a targeted literature review on a total of 10 disease-level characteristics across the four dimensions. These 10 characteristics were selected to provide a comprehensive understanding of similarities in key disease aspects, including disease patterns, severity, market dynamics, and resource demands, to help identify cancer analogs suitable for informing diagnosis, treatment, reimbursement, and policy strategies for PAH. Each disease-level characteristic was measured using extracted values of one or more corresponding metrics (Table 1). See Table S2 for the specific rationale for disease-level characteristics and the reference characteristic values for PAH.

### 2.4. Targeted Literature Review and Data Extraction

A targeted literature review was conducted to extract data on disease-level characteristics of PAH and the cancer analogs using assessments from the Institute for Clinical and Economic Review (ICER) [18], supplemented by peer-reviewed and gray literature sources identified on PubMed, Cochrane Database of Systematic Reviews, Scopus, and Google Scholar databases. Examples of keyword search terms are displayed in Table S3. Literature searches adopted a pearl-growing approach to identify additional literature from reference lists of articles of interest and forward-searching of citations (see the Supplemental Materials for details) [19]. More details on the targeted literature review methodology are available in the Supplemental Materials.

### 2.5. Evaluation of Cancer Analogs Most Similar to PAH

The similarity of each cancer analog to PAH was determined based on the absolute difference between the cancer analog characteristic values and those of PAH. For instance, if the average age of onset for PAH is 40 and for skin melanoma is 65, the absolute difference is |40–65| = 25. The cancer analog with a numerical characteristic value closest to PAH (i.e., smallest absolute difference from the PAH value) was determined as the “most similar” cancer analog under that characteristic. Because clinical severity can vary widely by tumor stage, a sensitivity analysis was conducted using survival and mortality characteristics of cancer analogs specific to the regionalized stage for comparison to PAH. The results of the similarity analysis were organized into a summary table ranking the similarity of cancer analogs to PAH under each disease-level characteristic. All data collection and analyses were performed in Microsoft Excel (version 2502).

## 3. Results

**Figure 1:**
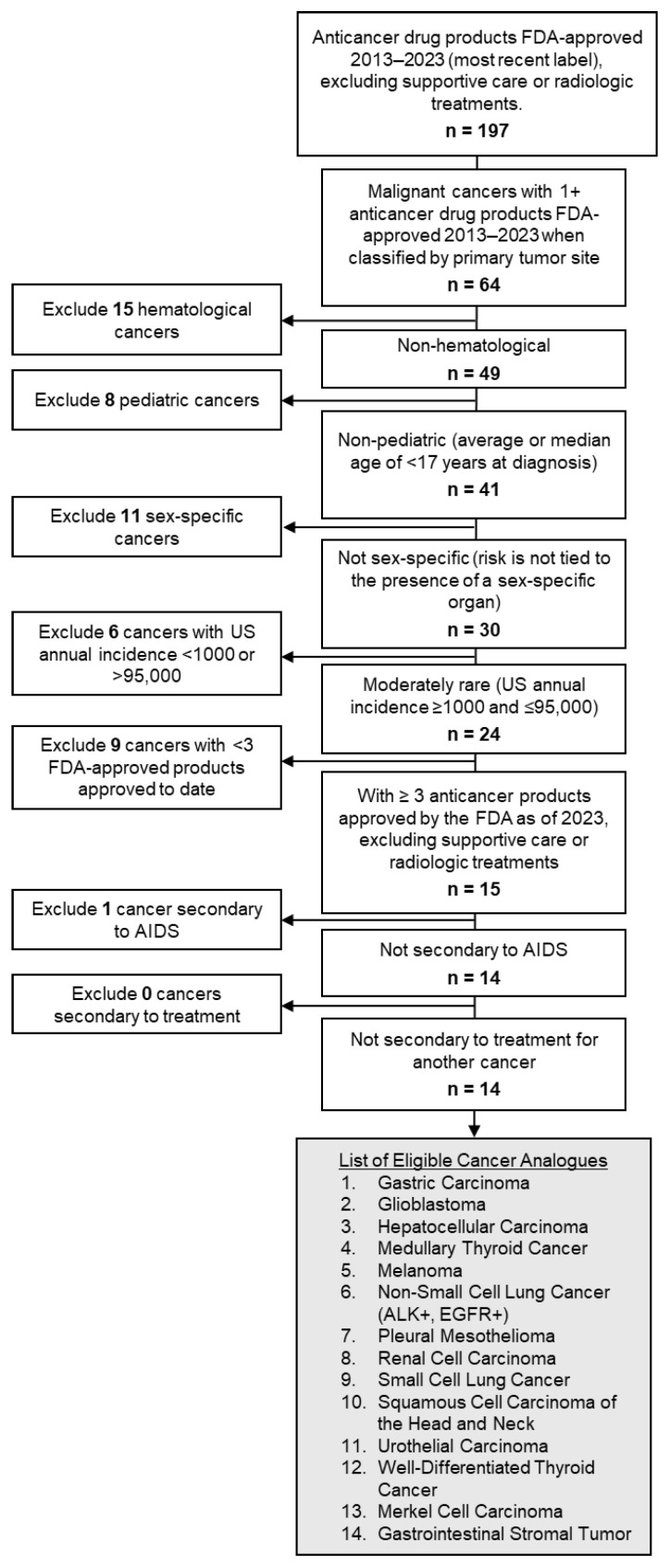
Potential cancer analogs selection results.

Out of 64 cancers with 2013–2023 FDA-approved pharmaceutical treatments, 14 cancers met the criteria for comparison to PAH (Figure 1). Cancer analogs in the sample were most often respiratory (*n* = 3; non-small-cell lung cancer (NSCLC), small cell lung cancer (SCLC), pleural mesothelioma) and digestive (*n* = 3; gastric carcinoma, gastrointestinal stromal tumor (GIST), hepatocellular carcinoma), followed by endocrine (*n* = 2; medullary thyroid cancer, well-differentiated thyroid cancer (WDTC), urinary (*n* = 2; renal cell carcinoma, urothelial carcinoma), skin (*n* = 2; skin melanoma, Merkel cell carcinoma), head and neck (*n* = 1; squamous cell carcinoma of head and neck), and central nervous system (*n* = 1; glioblastoma). Anaplastic lymphoma kinase-positive (ALK+) and epidermal growth factor receptor-positive (EGFR+) variants of NSCLC were evaluated separately.

**Figure 2:**
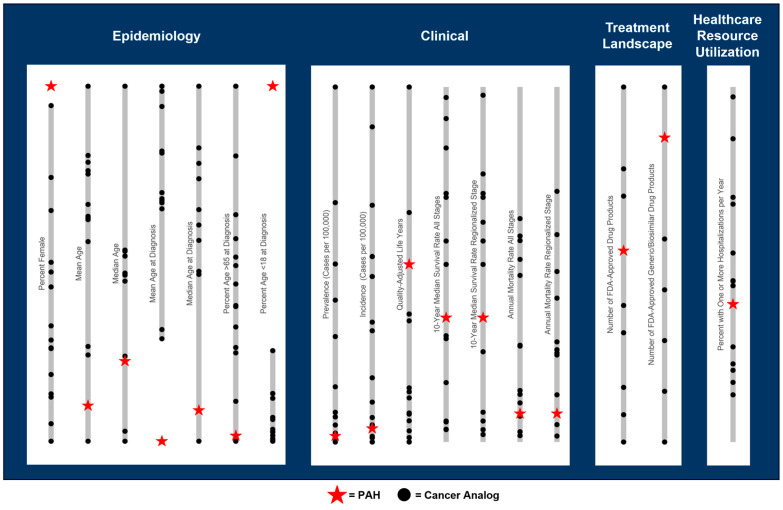
Overview of PAH characteristics relative to range across cancer analogs.

Characteristics of PAH generally overlapped with the characteristic values observed across the cancer analogs, particularly for clinical, therapeutic landscape, and HCRU dimensions (Figure 2). However, no single cancer analog ranked closest to PAH across all dimensions. Among the 14 cancers that met the selection criteria, WDTC had the highest number of characteristic metrics (*n* = 5) under which it ranked closest to PAH, followed by GIST (*n* = 4) (Table 1, Tables S4 and S5). The characteristics where WDTC was most similar to PAH included female prevalence of 76% (vs. 79% for PAH) [20,21], mean age of 54 (vs. 56) [4,22], median age at diagnosis of 51 (vs. 53) [23,24], proportion of patients aged 65+ at diagnosis of 23% (vs. 24%) [23,25], and disability-adjusted life years (DALYs) of 11.20 (vs. 2.56) [26,27,28]. Despite these demographic similarities, WDTC is much more common than PAH, with a US prevalence per 100,000 of 296.8 vs. 9.3 for PAH [22,29]. WDTC is also slower to progress, as exhibited by a substantially higher 10-year survival rate of 97% across all stages and 98% in the regionalized stage vs. 35% for PAH [5,22,30], despite a relatively similar annual mortality rate of 2% vs. 8% for PAH [4,22].

The cancer analog most similar to PAH varied across the four dimensions of characteristics considered (Table 2). Cancer analogs ranking in the top 3 closest to PAH under multiple disease characteristics within each dimension are described, as well as lung cancer analogs with notable similarities to PAH. With respect to epidemiology, the prevalent PAH population was generally more female and younger than the cancer analog populations (Figure 2), comprising 76% female patients and a mean (median) age of 56 (60) years [4,24]. PAH patients also tended to be diagnosed younger, with a mean (median) age of 40 (53) in the incident population [24,31], among which 15% were under the age of 18 at diagnosis [32], and 24% were over 65 [25]. While WDTC was most similar to PAH in epidemiological characteristics, medullary thyroid cancer was the second most similar, with 60% female patients, a mean (median) age of diagnosis of 50 (51) years, and 3.8% of patients diagnosed under 18 and 30.8% diagnosed over 65 (Table S6, Figure S1) [33,34,35]. Among lung-related cancer analogs, ALK+ NSCLC was also notably similar to PAH in terms of demographics, with a relatively young patient population (mean and median age: 60) [36], and a relatively lower proportion of patients diagnosed over 65 (46%) [37], although only 52% of patients were female [36].

With respect to clinical characteristics, PAH was relatively rare compared to most cancer analogs, with annual US incidence and prevalence of 1.25 and 9.3 per 100,000, respectively [29,38], ranking as the fourth lowest incidence and prevalence when compared across the 15 cancer analogs evaluated (14 cancers, with ALK+ and EGFR+ NSCLC evaluated separately). PAH was also associated with a higher quality of life as measured by the total quality-adjusted life years (QALYs) under standard of care (SoC) (4.0), ranking as the third highest among analogs [39,40,41]. However, PAH also exhibited notably comparable survival/mortality to the cancer analogs, even when restricting cancer analogs to the regionalized tumor stage (Figure 2). Compared across the cancer analogs, PAH’s annual mortality rate (8%) [4] ranked 9th highest, while 10-year (35%) [5] ranked 11th highest. GIST had the closest annual US incidence to PAH (1.5 per 100,000) [42] and a relatively similar prevalence (12.9 per 100,000) [43], while ALK+ NSCLC had the closest prevalence to PAH of 9.9 per 100,000 [44], also with a similar incidence of 2.0 per 100,000 [45] (Table S7, Figure S2). Across all tumor stages, GIST had the most similar annual mortality to PAH (7.3%) [46], while hepatocellular carcinoma was most similar in 10-year survival (30%) [47]. Restricting to the regionalized stage, annual mortality and 10-year survival were closest for skin melanoma (4.9%) and gastric carcinoma (25.4%), respectively [22]. Urothelial carcinoma was also similar to PAH in average QALYs under SoC (4.9) [48]. Compared to the lung-related cancer analogs, PAH generally had a better prognosis, with a 10-year survival and mortality rate closer to ALK+ NSCLC (17% and 27% across all stages, respectively) [49,50] versus other lung cancer analogs.

With respect to the therapeutic landscape, PAH had a similar number of FDA-approved pharmaceutical therapies compared to the cancer analogs (10), ranking seventh highest across cancer analogs, but the second highest number of pharmaceutical therapies with generic/biosimilar versions available (6) (Table S8, Figure S3) [17]. ALK+ NSCLC and renal cell carcinoma were most similar to PAH across therapeutic landscape characteristics. Like PAH, ALK+ NSCLC had 10 unique drug products on the US market at the time of data collection, but only 1 of these products had generic/biosimilar versions available [17]. Renal cell carcinoma had the closest number of drug products with generic/biosimilar alternatives to PAH (7) [17]. Among lung-related cancer analogs, EGFR+ NSCLC was also relatively similar to PAH in the unique number of drug products (13) and the number with generic/biosimilar alternatives (4) [17].

With respect to HCRU, the proportion of PAH patients hospitalized per year (38.8%) [51] was comparable to the cancer analogs, ranking ninth highest across analogs (Table S9, Figure S4). GIST was closest to PAH in HCRU, with 44.0% of patients hospitalized annually [52]. Among lung-related cancer analogs, PAH’s annual proportion of hospitalized patients was also relatively similar to EGFR+ NSCLC (26.8%) [53]. Detailed results and supporting literature are available in the Supplemental Materials [54,55,56,57,58,59,60,61,62,63,64,65,66,67,68,69,70,71,72,73,74,75,76,77,78,79,80,81,82,83,84,85,86,87,88,89,90,91,92,93,94,95,96,97,98,99,100,101,102,103,104,105,106,107,108,109,110,111,112,113,114,115,116,117,118,119,120,121,122,123,124,125,126,127,128,129,130,131,132,133,134,135,136,137,138].

## 4. Discussion

This study demonstrated that PAH shares key similarities to cancer with respect to demographics, disease severity, US therapeutic landscape, and resource utilization. No single cancer was identified as the best analog to PAH across all disease characteristics compared, highlighting the multidimensionality of PAH. However, characteristic values for PAH overlapped with the range of characteristics across the cancer analogs, with PAH ranking near the middle of the range of cancer analog characteristics, including annual mortality, 10-year survival, number of unique FDA-approved pharmaceutical therapies, and proportion of patients hospitalized per year, emphasizing the overall similarity of PAH to cancer.

Beyond similarities across these four dimensions, current studies also suggest that severe PAH shares several cancer-like biological and pathophysiological features. These include sustained proliferative signaling, the ability of cells to bypass growth-inhibitory signals, resistance to apoptosis, deregulation of cellular energetics, and the acquisition of replicative capacity and genomic instability driven by genetic and epigenetic alterations, alongside the activation of specific intracellular signaling cascades. Both PAH and cancer are characterized by chronic inflammatory activation, pathological angiogenesis, and the capacity of cells to evade immune surveillance [7,139,140,141]. In addition, PAH is increasingly recognized as a systemic biological disorder. Growing evidence demonstrates that PAH has similar mitochondrial dysfunction, metabolic reprogramming, and bone-marrow-derived progenitor cell abnormalities as cancer, further supporting that PAH is a cancer-like disease [7,142].

Cancer awareness is widespread, supported by initiatives such as Breast Cancer Awareness Month in October [143], while popular films like *The Fault in Our Stars* bring personal cancer stories to mainstream audiences [144]. Fundraising efforts include high-profile media events like the Stand Up to Cancer telethon and partnerships with corporations like the US National Football League’s Crucial Catch campaign [145,146]. Individuals with cancer can also receive benefits such as free patient navigation services through the American Cancer Society [147]. There are over 10 major scientific conferences held in the US focused exclusively on cancer and even specific tumor types that draw thousands of participants annually. Throughout history, physician-led initiatives such as the Jimmy Fund, established by Dr. Sidney Farber for childhood cancer research, have been instrumental in increasing public awareness of cancer and influencing policy changes [148].

Raising awareness is crucial for improving outcomes in PAH, a condition often underrecognized due to its rarity and non-specific presentation [149]. Lessons from public health strategies targeting other diseases suggest that linking less well-known conditions to more familiar ones can improve awareness and outcomes; for instance, public health campaigns on HIV/AIDS have raised awareness of hepatitis C due to their similar transmission routes, contributing to increased rates of screening and cleared infection [150]. Similarly, the parallels between PAH and cancer highlighted in this study present an opportunity to leverage cancer’s widespread recognition to improve understanding of PAH’s severity and urgency among clinicians. By framing PAH in a manner similar to cancer, where early detection is known to significantly influence outcomes, general pulmonologists may prioritize earlier screening and comprehensive evaluation, such as through referrals for an echocardiogram, reducing diagnostic delays, and improving patient outcomes. Analogies to cancer may also help clinicians communicate with patients and caregivers about the serious nature of PAH, emphasizing the importance of timely diagnosis, regular monitoring, and optimized treatment to slow disease progression.

Recognizing the similarities between PAH and cancers may also have practical implications for clinical management and therapeutic innovation. Viewing PAH as a comparable disease to many cancers may support continued exploration of multi-targeted therapeutic interventions or the induction–maintenance concept, similar to oncology treatments, to prevent irreversible vascular remodeling. Specifically, our study shows that ALK+ non-small-cell lung cancer was most similar to PAH in terms of therapeutic landscape, suggesting that PAH is already being treated as a complex condition involving multiple biological pathways.

Furthermore, cancer comparisons can aid payers and policymakers in recognizing the significant burden and complexity of PAH. Given PAH’s reliance on specialized prognostic indicators and resource-intensive management, drawing parallels with cancer—a similarly complex and high-burden condition—may facilitate more informed decisions regarding coverage, reimbursement, and funding for PAH care and treatment.

## 5. Limitations

This study had several limitations. First, to feasibly collect a wide range of evidence across various diseases, literature reviews were targeted rather than systematic, meaning no individual parameter is definitive in nature. Second, two new therapies—sotatercept and macitentan/tadalafil combination—were approved by the FDA for treatment of PAH since the data collection for this study was completed, thus they were not included in therapeutic landscape comparisons. Third, although the cancer analogs can be further broken down into subgroups based on prognostic stage and line of therapy, across which the collected characteristics may vary, the study prioritized more general disease and burden comparisons to PAH over clinical precision. Fourth, while this study did not create a single numerical index to measure similarity across all the considered dimensions, future research should explore this method in applications tailored to specific stakeholders, weighting characteristics based on their relative importance to the audience and context. Despite these limitations, the key strength of this study was the breadth of evidence collected and synthesized across a variety of cancers to systematically identify similarities between PAH and cancers based on empirical comparison of their disease characteristics.

## 6. Conclusions

This study employed an empirical approach to show that PAH shares key similarities to various cancers beyond molecular mechanisms, in terms of disease severity, resource utilization, and therapeutic landscape characteristics. While no single cancer analog was identified as the “perfect” analog for PAH, the characteristics of PAH overlapped with a range of characteristics across the cancer analogs, highlighting the similarity of PAH to various types of cancers, as well as the multidimensionality of PAH, particularly given the distinct differences in characteristics between the types of cancer. The cancer analogs identified in this study can serve as a useful tool to increase recognition and understanding of PAH to help improve outcomes in this high-risk population. To general pulmonologists and other clinicians, clinical similarities to cancer analogs identified, such as ALK+ and EGFR+ NSCLC, can help to communicate PAH’s severe and progressive nature, emphasize the importance of early diagnosis and treatment, and advocate for increased awareness. To resource allocation decision-makers, such as policymakers, research funding administrators, and payers, HCRU and market landscape similarities to ALK+ and EGFR+ NSCLC can help to justify the allocation of funding for the effective management of PAH and promote research on treatment innovations, as in the oncology space.

## Figures and Tables

**Table 1:** Disease-level characteristics and corresponding metrics.

Dimension	Disease-Level Characteristic	Disease-Level Metric	Definition of Data Values
Epidemiological	Sex	Prevalence of females	Percentage (%) of female patients.
	Age	Mean age	Mean age of prevalent population.
		Median age	Median age of prevalent population.
	Age at diagnosis	Mean age at diagnosis	Mean age of incident population.
		Median age at diagnosis	Median age of incident population.
		Aged < 18 years at diagnosis	Percentage (%) of incident population aged < 18 years.
		Aged 65+ years at diagnosis	Percentage (%) of incident population aged > 65 years.
Clinical	Prevalence/Incidence	Prevalence rate	US prevalence measures as number of cases per 100,000 population, reported as a percentage (%).
		Annual incidence rate	US annual incidence measures as the number of newly diagnosed patients with the analog disease per 100,000 people and reported as a percentage (%).
	Survival/Mortality	10-year median survival rate	10-year median survival rate, reported as a percentage (%). If 10-year median survival was unavailable, 5-year or 1-year median survival was considered.
		Annual mortality rate	Annual mortality rate, reported as a percentage (%).
		Median time from diagnosis to death	Median time from diagnosis to death, reported in months.
	Functional status	Functional status scale	Binary indicator: value = 1, if there is a functional status scale or holistic staging framework for the disease; value = 0, if otherwise.
		Mobility problem	Binary indicator: value = 1 if mobility problems are a prominent sequela of the disease; value = 0 otherwise.
	Quality of life	Quality-adjusted life years (QALYs)	Average total lifetime QALYs, which quantifies the health effect under standard of care (SoC) treatment.
		Disability-adjusted life years (DALYs)	Average number of DALYs, defined as the number of years of “healthy” life lost to the disease.
Therapeutic Landscape	Unique products	Unique products available	Number of unique products (based on active moiety, irrespective of mode of delivery) indicated for the disease on the US market.
	Generics/Biosimilars	Number of generic/biosimilar products available	Number of unique products (based on active moiety, irrespective of mode of delivery) with at least one generic/biosimilar product on the US market.
Healthcare Resource Utilization	Hospitalization	Annual hospitalization rate	Percentage (%) of patients with the disease who have at least one all-cause hospitalization in a given year.

**Table 2:** Cancer analogs ranking in the top 3 most similar to PAH by disease-level metric.

Comparison Dimension	Disease-Level Characteristic	Disease-Level Metric	PAH Similarity Rank Under Disease-Level Metric		
			1 (Most Similar to PAH)	2	3
Epidemiological	Sex	Prevalence of females	Well-differentiated thyroid cancer	Non-small-cell lung cancer (NSCLC), EGFR+	Medullary thyroid cancer
	Age	Mean age	Well-differentiated thyroid cancer	Glioblastoma	Non-small-cell lung cancer (NSCLC), ALK+
		Median age	Gastrointestinal stromal tumor (GIST)	Non-small-cell lung cancer (NSCLC), ALK+	Glioblastoma
	Age at Diagnosis	Mean age at diagnosis	Medullary thyroid cancer	Non-small-cell lung cancer (NSCLC), ALK+	Well-differentiated thyroid cancer
		Median age at diagnosis	Medullary thyroid cancer	Well-differentiated thyroid cancer	Gastrointestinal stromal tumor (GIST)
		Aged 65+ years at diagnosis	Well-differentiated thyroid cancer	Medullary thyroid cancer	Hepatocellular carcinoma
		Aged <18 years at diagnosis	Medullary thyroid cancer	Gastrointestinal stromal tumor (GIST)	Well-differentiated thyroid cancer
Clinical	Prevalence/ Incidence	Prevalence rate	Non-small-cell lung cancer (NSCLC), ALK+	Small cell lung cancer	Glioblastoma
		Annual incidence rate	Gastrointestinal stromal tumor (GIST)	Pleural mesothelioma	Merkel cell carcinoma
	Survival/ Mortality	10-year median survival rate	Hepatocellular carcinoma	Gastric carcinoma	Merkel cell carcinoma
		Annual mortality rate	Gastrointestinal stromal tumor (GIST)	Urothelial carcinoma	Melanoma (Skin)
		Median time from diagnosis to death	Urothelial carcinoma	Non-small-cell lung cancer (NSCLC), ALK+	Gastric carcinoma
	Quality of Life	QALYs under SoC	Renal cell carcinoma	Urothelial carcinoma	Melanoma (Skin)
		DALYs under SoC	Well-differentiated thyroid cancer	Pleural mesothelioma	Melanoma (Skin)
Therapeutic Landscape	Unique Products	Number of unique products available	Non-small-cell lung cancer (NSCLC), ALK+	Hepatocellular carcinoma	Urothelial carcinoma
	Generics/ Biosimilars	Number of generic/biosimilar products available	Renal cell carcinoma	Non-small-cell lung cancer (NSCLC), EGFR+	Gastric carcinoma
Healthcare Resource Utilization	Hospitalization	Annual hospitalization rate	Gastrointestinal stromal tumor (GIST)	Merkel cell carcinoma	Squamous cell carcinoma of head and neck (H&N)

Note: Functional status scale and mobility disease-level characteristics can only take the value of 0 or 1, and so, they are not displayed in the top 3 ranking table. Abbreviations: DALYs—disability-adjusted life years; PAH—pulmonary arterial hypertension; SoC—standard of care; QALYs—quality-adjusted life years.

## Data Availability

The original contributions presented in this study are included in the article/Supplementary Materials. Further inquiries can be directed to the corresponding author(s).

## Supplementary Materials

The following supporting information can be downloaded at: https://www.mdpi.com/article/10.3390/jmahp14010009/s1, Table S1: Cancer analogs selection criteria, Table S2: PAH reference values and rationale for disease-level characteristics, Table S3: Sample search terms, Table S4: PAH similarity rankings by disease-level characteristic—Epidemiological and clinical dimensions, Table S5: PAH similarity rankings by disease-level characteristic—Treatment landscape and health care resource utilization dimensions, Table S6: Data collected on epidemiological disease-level characteristics, Table S7: Data collected on clinical disease-level characteristics, Table S8: Data collected on therapeutic landscape disease-level characteristics, Table S9: Data collected on healthcare resource utilization disease-level characteristics, Figure S1: Distribution of cancer analogs within each epidemiology disease-level metric, Figure S2: Distribution of cancer analogs within each clinical landscape disease-level metric, Figure S3: Distribution of cancer analogs within each treatment landscape disease-level metric, Figure S4: Distribution of cancer analogs within each healthcare utilization landscape disease-level metric.

## Abbreviations

The following abbreviations are used in this manuscript:

PAH	Pulmonary arterial hypertension
HCRU	Healthcare resource utilization
ALK+	Anaplastic lymphoma kinase-positive
PH	Pulmonary hypertension
FDA	Food and Drug Administration
US	United States
ICER	Institute for Clinical and Economic Review
NSCLC	Non-small-cell lung cancer
SCLC	Small cell lung cancer
GIST	Gastrointestinal stromal tumor
WDTC	Well-differentiated thyroid cancer
EGFR+	Epidermal growth factor receptor-positive
DALYs	Disability-adjusted life years
QALYs	Quality-adjusted life years
SoC	Standard of care
H&N	Head and neck
