# Supplementary material for: Pulmonary Arterial Hypertension and Cancer: Unveiling Parallels in Epidemiology, Clinical Pathways, and Therapeutic Strategies
Source: J Mark Access Health Policy. 2026 Feb 6;14(1):9. doi: 10.3390/jmahp14010009 (PMC12921831; doi:10.3390/jmahp14010009)
Supplement: Supplementary file 1 [file jmahp-14-00009-s001.zip › jmahp-4053375-supplementary.pdf]

Supplemental Materials

All references cited in the Supplementary Materials are included in the main reference list; reference numbers correspond to the main reference list.

Pulmonary Arterial Hypertension and Cancer: Unveiling Parallels in Epidemiology, Clinical Pathways, and Therapeutic Strategies

Contents

Supplemental Methods 2
- Rationale for Cancer Selection Criteria 2
- Rationale for Comparison Dimensions and Disease-Level Characteristics 3
- Targeted Literature Review 6

Supplemental Results 8

Supplemental Methods

Rationale for Cancer Selection Criteria

Inclusion and exclusion criteria for selection of potential cancer analogs for comparison to PAH is presented in **Table S1**Error! Reference source not found..

Given that PAH is a rare but not ultra-rare disease with a US annual incidence rate of 4,163 [38], the study limited the cancer analogs to moderately rare cancers with a US annual incidence rate ⩾1,000 and ⩽95,000. Lower US incidence bound of 1,000 was based on the upper threshold of FDA's ultra rare disease definition [8] to include relative rare but not ultra-rare cancers. The upper incidence bound of 95,000 was based on an estimated annual incidence rate of pulmonary hypertension [9] to exclude very common cancers which may be poor analogs to PAH and include sufficiently common cancers to increase likelihood of familiarity.

Hematological cancers were excluded given that they have unique staging and are classified as "distant" cancers by the National Cancer Institute, which indicates that the cancer has reached distant parts of the body, while PAH is regionalized to the pulmonary system. Cancers that are pediatric-onset were excluded due to the differences in disease progression, prognosis, and treatment options compared to the adult population. Cancers whose risk is tied to the presence of a sex-specific organ, such as breast cancer and prostate cancer, were excluded given that both males and females are at risk for PAH. Cancers secondary to AIDS such as Kaposi sarcoma have unique epidemiology that would make them a poor analog for PAH and were therefore excluded. Cancers that are secondary to other treatments are strongly heterogenous due to the manner of treatment, such as age of treatment, dosage, and area and tissue treated, and were therefore excluded.

Table S1. Cancer Analogs Selection Criteria

Inclusion Criteria
**Recent drug approval**. Cancers with at least one (1) anticancer product approved by the FDA between 2013-2023 (using the most recent label date), excluding palliative, adjuvant, and radiologic treatments.
**Multiple treatments available**. Cancers with at least three (3) anticancer products approved by the FDA as of 2023, excluding supportive care therapies or radiologic treatments
**Moderately rare disease**. Cancers with an annual incidence rate of less than or equal to 95,000 and greater than or equal to 1,000 individuals in the U.S.
**Exclusion Criteria**
**Hematological cancers**. Cancers that are not classified as hematological cancers.
**Pediatric-onset cancers**. Cancers that do not have an average or median age of <17 years at diagnosis, in accordance with the FDA's definition of pediatric cancer
**Sex-specific cancers.** Cancers whose risk is not tied to the presence of a sex-specific organ (e.g., excludes breast cancer, penile cancer, prostate cancer, testicular cancer, cervical cancer, ovarian cancer, uterine cancer, vaginal cancer, vulval cancer).
**Secondary to AIDS.** Cancers that are not secondary to AIDS (e.g., Kaposi sarcoma)
**Secondary to treatment.** Cancers that are not secondary to treatment for another cancer (e.g., Myelodysplastic syndrome subsequent to chemotherapy)

Rationale for Comparison Dimensions and Disease-Level Characteristics

Disease level characteristics were collected across epidemiological, clinical, therapeutic landscape, and healthcare resource utilization (HCRU) dimensions for comparison to PAH (**Table S2**).

The epidemiological dimension evaluated the similarity of patient populations with respect to distributions of sex, age, and age at diagnosis. Age and sex can point to similarity in epidemiological patterns and biological pathways between diseases while age at diagnosis can provide insight into similarity of disease trajectory. PAH is more common among women and usually occurs at mid-life [4,21]; diseases with similar demographic profiles can reflect similarity in the risk profile of these patients, which can inform the development of insurance premium structures and pricing.

The clinical dimension evaluated the similarity of clinical profiles in terms of US prevalence, survival/mortality rate, functional status, and quality of life. PAH is a rare, severe, and progressive disease that leads to significant functional impairment and mortality [3,16]. Comparison of prevalence/incidence ensures that disease analogs are similarly rare to PAH, which can represent shared challenges with diagnosis and incentives for treatment development such as orphan drug designation. Diseases with similar survival/mortality, functional status, and quality of life likely have similar patient needs and comparable costs associated with medical care, long term support, and loss of productivity, which can inform reimbursement and resource allocation policies.

The therapeutic landscape dimension evaluated similarity in terms of availability of unique FDA-approved (based on active moiety, irrespective of mode of delivery) treatments and number of generic or biosimilar equivalents on the US market. Although PAH has no cure, multiple treatments are available to slow progression and manage symptoms [11]. Disease analogs with a similar market presence can serve as models for developing reimbursement and policy strategies tailored to PAH. The number of unique products available for a disease reflects the competitive market dynamics, level of innovation, and potential for novel and breakthrough therapies, informing payor bargaining power for reimbursement policies. Similar availability of generics and biosimilars can help to forecast the financial implications of coverage decisions.

The HCRU dimension evaluated similarity of economic burden in terms of hospitalization rates. Approximately one in three patients with PAH is hospitalized per year [51]. Similar hospitalization rates can indicate a similar level of resource utilization required for patient care.

Table S2. PAH Reference Values and Rationale for Disease-Level Characteristics

Dimension	Disease-Level Characteristic	Disease-Level Metric	PAH Reference Values	Rationale
**Epidemiological**	Sex	Prevalence of females	79% of STELLAR trial participants with PAH were female [21]	To characterize the sex distribution of the current patient population of the disease analog, given that a majority of PAH patients are female.
	Age	Mean age	Average age of PAH patients was 57 years old [4,12]	To characterize the age distribution of the current prevalent disease analog population.
		Median age	Median age of PAH patients was 60 years old [4]	
	Age at Diagnosis	Mean age at diagnosis	Average age at diagnosis for PAH was roughly between 50-65 years old [5,21]	To characterize the age distribution of the current incident disease analog population.

Dimension	Disease-Level Characteristic	Disease-Level Metric	PAH Reference Values	Rationale
		Median age at diagnosis	Median age at diagnosis for PAH was 53 years old [24]	
		Aged <18 years at diagnosis	14.9% of patients were diagnosed under 18 years old [32]	
		Aged 65+ years at diagnosis	23.9% of patients were diagnosed over 65 years old [25]	
**Clinical**	Prevalence/Incidence	Prevalence rate	The prevalence rate for PAH, reported in 2016 was 9.3 cases per 100,000 [29]	To characterize the size of the current US disease analog population.
		Incidence rate	The annual incidence of PAH in the U.S. is estimated to be roughly 1-1.5 cases per 100,000 [11,13,38]	To characterize the size of the newly diagnosed US disease analog population.
	Survival / Mortality	10-year median survival rate	The 10-year median survival rate for PAH patients is 35% [5]	To measure the expected survival of patients with the disease analog. 10-year median survival was the primary data point of interest; if unavailable, 5-year or 1-year median survival rates were considered. If survival endpoints were collected at different time points, a proportional hazard assumption was applied to estimate the median survival to facilitate comparison across a diverse set of diseases. Annual mortality rate and median time from diagnosis to death metrics was additionally collected.
		Annual mortality rate	Annual mortality rate for PAH patients is 8% [4]	
		Median time from diagnosis to death	The median time from diagnosis to death for PAH patients was 6 years [54]	
	Functional Status	Functional status scale	PAH does have a functional status or classification scale, as defined by the World Health Organization (WHO) [14]	To determine whether the disease analog has a functional status scale/classification or functional status indicators (e.g., activities of daily living (ADLs) and instrumental activities of daily living (IADLs) that demonstrate whether patients can finish basic living tasks independently), given that PAH significantly impairs patients' ability.
		Mobility problem	PAH patients suffer from right heart failure, which	To examine whether mobility scales are widely used to

Dimension	Disease-Level Characteristic	Disease-Level Metric	PAH Reference Values	Rationale
			results in the limitation on physical activity [15]	evaluate symptom progression for that disease or whether mobility problems are noted as a prominent sequela for the disease, given that PAH has a large impact on mobility.
	Quality of Life	Quality-Adjusted Life Years (QALYs)	PAH patients who used standard care/treatments have QALYs of 4 [39-41]	To determine how disease analogs affect patient quality of life under standard of care treatment, measured via QALYs, which combines the impact of life duration and life quality, and DALYs, or the sum of years lost from life and years lost due to disability caused by the disease.
		Disability-Adjusted Life Years (DALYs)	PAH patients have 2.56 DALYs [27,28]	
**Therapeutic Landscape**	Unique Products	Unique Products Available	10 unique PAH products are available in the US market [17]	To characterize the competitive landscape faced by drug manufacturers and payor negotiating power.
	Generics / Biosimilars	Number of Generic / Biosimilar Products Available	6 (60%) of the 10 unique PAH products have at least one generic available on the US market [17]	To determine the impact of presence of generic or biosimilar treatment options in the drug manufacturer competitive landscape and on payor negotiating power. For Medicare beneficiaries, these metrics included physician-administered drugs (e.g., biologics) covered by Medicare Part B and outpatient drugs covered by Medicare Part D. For commercial plan patients, these metrics included physician-administered drugs covered by the medical benefit and drugs covered under by pharmacy benefit.
**Healthcare Resource Utilization**	Hospitalization	Annual Hospitalization Rate	The annual hospitalization rate for PAH was 38.8% [51]	To characterize the burden of the disease analog in terms of healthcare utilization.

Targeted Literature Review

A targeted literature review was conducted to collect data on disease-level characteristics of PAH and each potential disease analog meeting the selection criteria. Institute For Clinical and Economic Review (ICER) reports were the primary source of review because of their focus on recently-approved treatments and use of relatively up-to-date data on disease characteristics. In cases where ICER reports did not have information on a disease-level characteristic, targeted literature searches were conducted on PubMed, Cochrane Database of Systematic Reviews, Scopus (including all of MEDLINE and Embase) and Google Scholar databases to identify peer-reviewed literature sources. If a reliable US source was not available, global estimates or estimates from other developed countries were used. Peer-reviewed literature sources were identified for inclusion through keyword search terms related to the short-list disease analogs, the comparison dimensions, and the disease-level characteristics. Examples of keyword search terms corresponding to the potential disease analogs are displayed in **Table S3**. After identifying articles of interest, the literature search adopted a pearl-growing approach to identify additional literature via referenced studies and forward-searching of citations of abstracts and articles reviewed [19].

If data was unavailable in peer-reviewed literature, grey literature sources including conference abstracts/proceedings, white papers, patient advocacy publications, and physician opinion were identified through searches on Google Scholar, Google Search, Google News, and the Centers for Medicare & Medicaid Services (CMS) website. Structured product labels on the FDA label website were searched to determine the number and types of treatments available for the disease analogs [17].

Because this targeted literature review collected data on numerous disease-level characteristics for each analog disease, it primarily aimed for breadth of information. Given the large amount of information collected, source quality was evaluated qualitatively by the study team in place of a formal quality assessment procedure. When more than one source was identified with data on a disease-level metrics, the preferred source was chosen based on journal reputation, study sample size, study recency, study design, and concordance with previous literature.

Table S3. Sample Search Terms

Dimension	Disease-Level Characteristic	Key Search Term
**Epidemiological**	Sex	"Proportion of patients female"<br>"Proportion of patients male"<br>"Sex composition"
	Age	"Age at diagnosis"<br>"Average patient age"<br>"Median patient age"
	Age at diagnosis	"Mean age at diagnosis"<br>"Median age at diagnosis"<br>"Percent of patients diagnosed over age 65"<br>"Percent of patients diagnosed under 18"
**Clinical**	Prevalence	"Prevalence"<br>"Incidence"
	10-Year median survival rate	"Expected survival"<br>"5-year survival"<br>"10-year survival"
	Annual mortality rate	"Annual mortality rate"<br>"1-year mortality rate"
	Median time from diagnosis to death	"Median time from diagnosis to death"<br>"Expected life years after diagnosis"<br>"Survival after disease onset"
	Functional status	"functional status scale"<br>"quality of life"<br>*[Addtionally searched ClinicalTrials.gov for functional status scales in Phase III trials of treatments]*
	Mobility problem	"mobility problem related with the disease"

Dimension	Disease-Level Characteristic	Key Search Term
	Quality adjusted life years	“Quality adjusted life years”<br>“QALY”<br>“Cost effectiveness model”<br>“Cost effectiveness analysis”<br>“Quality of life”
	Disability adjusted life years	“Disability adjusted life year”<br>“DALY”<br>“Cost effectiveness model”<br>“Cost effectiveness analysis”<br>“Quality of life”
**Therapeutic Landscape**	Available unique products	*N/A [based on data on FDA website]*
	Number of generic/biosimilar products	*N/A [based on data on FDA website]*
**Healthcare Resource Utilization**	Hospitalization rate	“Annual hospitalization rate”<br>“Hospitalization rate”<br>“Share of patients hospitalized”<br>“Claims data” “Hospitalization rate”

Supplemental Results

Table S4. PAH Similarity Rankings by Disease-Level Characteristic - Epidemiological and Clinical Dimensions

Disease Analogue Similarity Rank to PAH	Epidemiology							Clinical						
	Gender	Age (Mean Age)	Age (Median Age)	Age at Diagnosis (Mean Age)	Age at Diagnosis (Median Age)	Age at Diagnosis (% Over 65)	Age at Diagnosis (% Under 18)	Disease Prevalence *(per 100,000)*	Incidence Rate *(per 100,000)*	10-Year Median Survival Rate	Annual Mortality Rate	Median Time From Diagnosis To Death *(Months)*	Quality-Adjusted Life-Year (QALYs) of Standard Care	Disability-Adjusted Life Years (DALYs)
1	WDTC[1]	WDTC	GST	MTC	MTC	WDTC	MTC	NSCLC, ALK+	GST	HC	GST	UC	RCC	WDTC
2	NSCLC, EGFR+[2]	GBM	NSCLC, ALK+	NSCLC, ALK+	WDTC	MTC	GST	SCLC	PM	GC	UC	NSCLC, ALK+	UC	PM
3	MTC[3]	NSCLC, ALK+	GBM	WDTC	GST	HC	WDTC	GBM	MCC	MCC	MSC	GC	MSC	MSC
4	NSCLC, ALK+[4]	HC	SCCHN	RCC	HC	GST	GBM	GST	NSCLC, ALK+	GST	RCC	MTC	MTC	UC
5	SCLC[5]	RCC	WDTC	SCCHN	RCC	NSCLC, ALK+	RCC	PM	MTC	NSCLC, ALK+	WDTC	NSCLC, EGFR+	GC	GC
6	GST[6]	MSC	HC	GBM	SCCHN	SCCHN	HC	NSCLC, EGFR+	GBM	SCCHN	SCCHN	MSC	GBM	HC
7	MSC[7]	SCCHN	RCC	HC	GBM	GBM	SCCHN	HC	HC	PM	NSCLC, ALK+	GBM	NSCLC, EGFR+	GBM
8	PM[8]	NSCLC, EGFR+	MSC	MSC	NSCLC, EGFR+	NSCLC, EGFR+	MSC	GC	SCLC	NSCLC, EGFR+	MCC	PM	PM	MTC
9	GC[9]	SCLC	SCLC	GC	MCC	MSC	PM	MTC	GC	GBM	GC	HC	NSCLC, ALK+	NSCLC, EGFR+
10	GBM[10]	PM	PM	SCLC	MSC	RCC	GC	SCCHN	NSCLC, EGFR+	SCLC	PM	RCC	HC	NSCLC, ALK+
11	RCC[11]	GC	GC	UC	GC	SCLC	UC	RCC	SCCHN	RCC	GBM	SCLC	SCLC	RCC
12	HC[12]	UC	UC	MCC	SCLC	GC	SCLC	UC	WDTC	UC	HC	SCCHN	SCCHN	SCLC
13	SCCHN[13]	MCC	MTC	PM	UC	PM	MCC	WDTC	RCC	MTC	SCLC	WDTC	GST	SCCHN
14	MCC[14]	MTC	NSCLC, EGFR+	NSCLC, EGFR+	PM	UC	NSCLC, EGFR+	MSC	MSC	MSC	MTC	MCC	WDTC	MCC
15	UC[15]	GST	MCC	GST	NSCLC, ALK+	MCC	NSCLC, ALK+	MCC	UC	WDTC	NSCLC, EGFR+	GST	MCC	GST

Data points not identified for relevant disease (grey shading)

1. Well-Differentiated Thyroid Cancer
2. Non-Small Cell Lung Cancer, EGFR+
3. Medullary Thyroid Cancer
4. Non-Small Cell Lung Cancer, ALK+
5. Small Cell Lung Cancer
6. Gastrointestinal Stromal Tumor
7. Melanoma Skin Cancer
8. Pleural Mesothelioma
9. Gastric Carcinoma
10. Glioblastoma
11. Renal Cell Carcinoma
12. Hepatocellular
13. Squamous Cell Carcinoma of Head & Neck
14. Merkel Cell Carcinoma
15. Urothelial Carcinoma

Table S5. PAH Similarity Rankings by Disease-Level Characteristic - Treatment Landscape and Health Care Resource Utilization Dimensions

Disease Analog Similarity Rank to PAH	Treatment Landscape		Healthcare Resource Utilization	Regionalized Stage Sensitivity Analysis	
	Available Unique Products	Number of Generic/Biosimilar Products Available	Annual Hospitalization Rate	10-Year Median Survival Rate (Regionalized)	Annual Mortality Rate (Regionalized)
1	NSCLC, ALK+[4]	RCC	GST	GC	MSC
2	HC[12]	NSCLC, EGFR+	MCC	MCC	SCCHN
3	UC[15]	GC	SCCHN	GST	WDTC
4	GC[9]	GBM	NSCLC, EGFR+	SCCHN	MTC
5	GBM[10]	MSC	WDTC	HC	GC
6	NSCLC, EGFR+[2]	SCLC	MSC	MSC	RCC
7	SCLC[5]	SCCHN	SCLC	PM	UC
8	SCCHN[13]	WDTC	RCC	SCLC	SCLC
9	WDTC[1]	HC	GBM	GBM	PM
10	MSC[7]	NSCLC, ALK+	HC	RCC	HC
11	RCC[11]	PM	GC	UC	GBM
12	GST[6]	UC	PM	MTC	NSCLC, EGFR+
13	MTC[3]	GST	UC	WDTC	NSCLC, ALK+
14	PM[8]	MTC	MTC	NSCLC, EGFR+	MCC
15	MCC[14]	MCC	NSCLC, ALK+	NSCLC, ALK+	GST

(grey box) Data points not identified for relevant disease

1. Well-Differentiated Thyroid Cancer
2. Non-Small Cell Lung Cancer, EGFR+
3. Medullary Thyroid Cancer
4. Non-Small Cell Lung Cancer, ALK+
5. Small Cell Lung Cancer
6. Gastrointestinal Stromal Tumor
7. Melanoma Skin Cancer
8. Pleural Mesothelioma
9. Gastric Carcinoma
10. Glioblastoma
11. Renal Cell Carcinoma
12. Hepatocellular Carcinoma
13. Squamous Cell Carcinoma of Head & Neck
14. Merkel Cell Carcinoma
15. Urothelial Carcinoma

Table S6. Data Collected on Epidemiological Disease-Level Characteristics

Disease-Level Characteristic	Dimension Raw Values and Data Attributes	Disease Name	Raw Data Value	Reference
**Sex**	The most recently-available percentage (%) of female patients with the analog disease.	Urothelial Carcinoma	24.2%	[55]
		Merkel Cell Carcinoma	26.9%	[56]
		Squamous Cell Carcinoma of H&N	31.0%	[57]
		Hepatocellular Carcinoma	31.5%	[58]
		Renal Cell Carcinoma	34.5%	[59]
		Glioblastoma	38.5%	[60]
		Gastric Carcinoma	38.6%	[61]
		Pleural Mesothelioma	39.8%	[22]
		Melanoma (Skin)	42.0%	[62]
		Gastrointestinal Stromal Tumor	48.0%	[46]
		Small Cell Lung Cancer	50.3%	[63]
		NSCLC, ALK+	51.8%	[36]
		Medullary Thyroid Cancer	59.8%	[35]
		NSCLC, EGFR+	64.9%	[64]
		Well-Differentiated Thyroid	76.0%	[20]
		**Pulmonary Arterial Hypertension**	**79.0%**	**[21]**
**Mean Age of Patients**	The most recently-available average age for patients with the analog disease (age of prevalent population).	Medullary Thyroid Cancer	Not Found	
		Gastrointestinal Stromal Tumor	Not Found	
		Well-Differentiated Thyroid	53.9	[22]
		**Pulmonary Arterial Hypertension**	**56.0**	**[4]**
		Glioblastoma	59.0	[22]
		NSCLC, ALK+	59.5	[36]
		Hepatocellular Carcinoma	65.7	[22]
		Renal Cell Carcinoma	67.0	[22]
		Melanoma (Skin)	67.1	[22]
		Squamous Cell Carcinoma of H&N	67.2	[22]
		NSCLC, EGFR+	67.9	[65]
		Small Cell Lung Cancer	69.7	[22]
		Pleural Mesothelioma	69.9	[22]
		Gastric Carcinoma	70.4	[22]
		Urothelial Carcinoma	70.8	[66]
		Merkel Cell Carcinoma	74.9	[67]
**Median Age of Patients**	The most recently-available median age for patients with the analog disease (age of prevalent population).	Medullary Thyroid Cancer	Not Found	
		NSCLC, EGFR+	Not Found	
		Merkel Cell Carcinoma	Not Found	
		Well-Differentiated Thyroid	52.0	[22]
		Glioblastoma	53.0	[68]
		**Pulmonary Arterial Hypertension**	**60.0**	**[24]**
		Gastrointestinal Stromal Tumor	60.0	[46]
		NSCLC, ALK+	60.5	[36]
		Squamous Cell Carcinoma of H&N	68.0	[22]
		Hepatocellular Carcinoma	68.6	[22]
		Renal Cell Carcinoma	68.7	[22]
		Melanoma (Skin)	68.8	[22]
		Small Cell Lung Cancer	70.5	[22]
		Pleural Mesothelioma	71.0	[22]
		Gastric Carcinoma	71.1	[22]
		Urothelial Carcinoma	87.5	[22]
		NSCLC, EGFR+	Not Found	

Disease-Level Characteristic	Dimension Raw Values and Data Attributes	Disease Name	Raw Data Value	Reference
**Age At Diagnosis, Average Age**	The most recently-available average age at which patients are diagnosed with the analog disease.	Gastrointestinal Stromal Tumor	Not Found	
		**Pulmonary Arterial Hypertension**	**40**	**[24,31]**
		Medullary Thyroid Cancer	50.1	[33]
		NSCLC, ALK+	51.0	[69]
		Well-Differentiated Thyroid	51.0	[70]
		Renal Cell Carcinoma	62.9	[22]
		Squamous Cell Carcinoma of H&N	63.5	[22]
		Glioblastoma	63.7	[22]
		Hepatocellular Carcinoma	63.9	[22]
		Melanoma (Skin)	64.5	[22]
		Gastric Carcinoma	68.4	[71]
		Small Cell Lung Cancer	68.6	[22]
		Urothelial Carcinoma	73.0	[55]
		Merkel Cell Carcinoma	74.5	[67]
		Pleural Mesothelioma	75.0	[22]
**Age At Diagnosis, Median Age**	The most recently-available median age at which patients are diagnosed with the analog disease (age of incidence).	NSCLC, ALK+	Not Found	
		Medullary Thyroid Cancer	51.0	[33]
		Well-Differentiated Thyroid	51.0	[23]
		**Pulmonary Arterial Hypertension**	**53.0**	**[24]**
		Gastrointestinal Stromal Tumor	61.8	[72]
		Hepatocellular Carcinoma	62.0	[73]
		Renal Cell Carcinoma	64.0	[22]
		Squamous Cell Carcinoma of H&N	64.0	[22]
		Glioblastoma	65.0	[22]
		NSCLC, EGFR+	65.0	[74]
		Merkel Cell Carcinoma	65.0	[75]
		Melanoma (Skin)	66.0	[22]
		Gastric Carcinoma	68.0	[22]
		Small Cell Lung Cancer	69.0	[22]
		Urothelial Carcinoma	70.0	[76]
		Pleural Mesothelioma	74.0	[22]
**Age At Diagnosis, % Over 65**	The most recently-available percentage (%) of patients whose age at diagnosis is over 65 years of age.	Well-Differentiated Thyroid	22.8%	[23]
		**Pulmonary Arterial Hypertension**	**23.9%**	**[25]**
		Medullary Thyroid Cancer	30.8%	[35]
		Hepatocellular Carcinoma	40.5%	[77]
		Gastrointestinal Stromal Tumor	41.6%	[72]
		NSCLC, ALK+	45.7%	[37]
		Squamous Cell Carcinoma of H&N	49.8%	[22]
		Glioblastoma	50.0%	[78]
		NSCLC, EGFR+	50.0%	[74]
		Melanoma (Skin)	54.3%	[22]
		Renal Cell Carcinoma	58.0%	[79]
		Small Cell Lung Cancer	59.7%	[80]
		Gastric Carcinoma	63.4%	[61]
		Pleural Mesothelioma	68.2%	[22]
		Urothelial Carcinoma	80.0%	[81]
		Merkel Cell Carcinoma	94.0%	[56]
**Age At Diagnosis, % Under 18**	The most recently-available	NSCLC, EGFR+	Not Found	
		NSCLC, ALK+	Not Found	
		Merkel Cell Carcinoma	0.0%	[56]

Disease-Level Characteristic	Dimension Raw Values and Data Attributes	Disease Name	Raw Data Value	Reference
	percentage (%) of patients whose age at diagnosis is less than 18 years of age.	Small Cell Lung Cancer	0.03%	[22]
		Gastric Carcinoma	0.1%	[22]
		Urothelial Carcinoma	0.1%	[22]
		Pleural Mesothelioma	0.2%	[22]
		Melanoma (Skin)	0.4%	[82]
		Squamous Cell Carcinoma of H&N	0.4%	[22]
		Hepatocellular Carcinoma	0.5%	[83]
		Renal Cell Carcinoma	0.9%	[22]
		Glioblastoma	1.0%	[22]
		Well-Differentiated Thyroid	1.8%	[84]
		Gastrointestinal Stromal Tumor	2.0%	[85]
		Medullary Thyroid Cancer	3.8%	[34]
		**Pulmonary Arterial Hypertension**	**14.9%**	**[32]**

Table S7. Data Collected on Clinical Disease-level Characteristics

Disease-Level Characteristic	Dimension Raw Values and Data Attributes	Disease Name	Raw Data Value	Reference	Notes
**Prevalence Rate**	The most recently-available prevalence of the disease in the U.S., defined as the number of patients with the analog disease per 100,000 people and reported as a percentage (%).	Merkel Cell Carcinoma	Not Found		
		Pleural Mesothelioma	1.9	[22]	1.9 Cases Per 100,000 Individuals
		Glioblastoma	6.3	[22]	6.3 Cases Per 100,000 Individuals
		Small Cell Lung Cancer	8.3	[22]	8.3 Cases Per 100,000 Individuals
		**Pulmonary Arterial Hypertension**	**9.3**	**[29]**	**9.3 Cases Per 100,000 Individuals**
		NSCLC, ALK+	9.9	[44,86]	9.9 Cases Per 100,000 Individuals
		Gastrointestinal Stromal Tumor	12.9	[43]	12.9 Cases Per 100,000 Individuals; Sweden
		NSCLC, EGFR+	22.8	[87]	22.8 Cases Per 100,000 Individuals
		Hepatocellular Carcinoma	32.8	[22]	32.8 Cases Per 100,000 Individuals
		Gastric Carcinoma	38.5	[88]	38.5 Cases Per 100,000 Individuals
		Medullary Thyroid Cancer	69.9	[22]	69.9 Cases Per 100,000 Individuals
		Squamous Cell Carcinoma of H&N	131.8	[22]	131.8 Cases Per 100,000 Individuals
		Renal Cell Carcinoma	176.7	[22]	176.7 Cases Per 100,000 Individuals
		Urothelial Carcinoma	221.2	[22]	221.2 Cases Per 100,000 Individuals
		Well-Differentiated Thyroid	296.8	[22]	296.8 Cases Per 100,000 Individuals
		Melanoma (Skin)	439.4	[22]	439.4 Cases Per 100,000 Individuals
**Incidence Rate**	The most recently-available incidence rate of the disease in the U.S. annually, defined as the number of patients with the analog disease per 100,000 people.	Medullary Thyroid Cancer	0.3	[89]	0.3 Cases Per 100,000 Individuals
		Merkel Cell Carcinoma	0.6	[90]	0.6 Cases Per 100,000 Individuals
		Pleural Mesothelioma	0.7	[22]	0.7 Cases Per 100,000 Individuals
		**Pulmonary Arterial Hypertension**	**1.25**	**[38]**	**1-1.5 Cases Per 100,000 Individuals**
		Gastrointestinal Stromal Tumor	1.5	[42]	1.5 Cases Per 100,000 Individuals
		NSCLC, ALK+	2.02	[45]	2.02 Cases Per 100,000 Individuals
		Glioblastoma	3.1	[22]	3.1 Cases Per 100,000 Individuals
		Hepatocellular Carcinoma	4.8	[91]	4.8 Cases Per 100,000 Individuals
		Small Cell Lung Cancer	4.8	[22]	4.8 Cases Per 100,000 Individuals
		Gastric Carcinoma	8.1	[88]	8.1 Cases Per 100,000 Individuals
		NSCLC, EGFR+	8.7	[22,87]	8.7 Cases Per 100,000 Individuals

Disease-Level Characteristic	Dimension Raw Values and Data Attributes	Disease Name	Raw Data Value	Reference	Notes
		Squamous Cell Carcinoma of H&N	11.9	[22]	11.9 Cases Per 100,000 Individuals
		Well-Differentiated Thyroid	13.3	[70]	13.3 Cases Per 100,000 Individuals
		Renal Cell Carcinoma	16.9	[22]	16.9 Cases Per 100,000 Individuals
		Melanoma (Skin)	22.4	[22]	22.4 Cases Per 100,000 Individuals
		Urothelial Carcinoma	25.2	[55]	25.2 Cases Per 100,000 Individuals
**10-Year Median Survival Rate**	The most recently-available 10-year median survival rate for patients diagnosed with the analog disease, reported as a percentage (%).	Small Cell Lung Cancer	3.5%	[80]	
		Glioblastoma	3.6%	[22]	
		NSCLC, EGFR+	5.66%	[49]	Extrapolated from 5-year survival rate; Japan
		Pleural Mesothelioma	5.90%	[22]	
		NSCLC, ALK+	16.7%	[49]	Extrapolated from 5-year survival rate
		Gastric Carcinoma	29.1%	[22]	
		Hepatocellular Carcinoma	29.9%	[47]	
		**Pulmonary Arterial Hypertension**	**35.0%**	**[5]**	
		Merkel Cell Carcinoma	50.0%	[75]	
		Gastrointestinal Stromal Tumor	50.0%	[72]	
		Squamous Cell Carcinoma of H&N	56.6%	[22]	
		Renal Cell Carcinoma	68.9%	[22]	
		Urothelial Carcinoma	70.0%	[81]	
		Medullary Thyroid Cancer	82.8%	[92]	Extrapolated from 5-year survival rate
		Melanoma (Skin)	91.1%	[22]	
		Well-Differentiated Thyroid	97.0%	[30]	Based on weighted average of individual stages
**Annual Mortality Rate**	The most recently-available annual mortality rate for patients diagnosed with the analog disease, reported as a percentage (%).	Medullary Thyroid Cancer	Not Found		
		NSCLC, EGFR+	Not Found		
		Well-Differentiated Thyroid	1.80%	[22]	Extrapolated by 1-year survival rate
		Melanoma (Skin)	2.9%	[22]	Extrapolated by 1-year survival rate
		Gastrointestinal Stromal Tumor	7.3%	[46]	Extrapolated by 1-year survival rate
		**Pulmonary Arterial Hypertension**	**8.0%**	**[4]**	
		Urothelial Carcinoma	11.0%	[22]	Extrapolated by 1-year survival rate
		Renal Cell Carcinoma	13.4%	[22]	Extrapolated by 1-year survival rate
		Squamous Cell Carcinoma of H&N	14.5%	[22]	Extrapolated by 1-year survival rate
		NSCLC, ALK+	27.0%	[50]	Extrapolated by 1-year survival rate
		Merkel Cell Carcinoma	27.2%	[90]	

Disease-Level Characteristic	Dimension Raw Values and Data Attributes	Disease Name	Raw Data Value	Reference	Notes
		Gastric Carcinoma	46.9%	[93]	Extrapolated by 1-year survival rate
		Pleural Mesothelioma	51.4%	[22]	
		Glioblastoma	56.7%	[22]	Extrapolated by 1-year survival rate
		Hepatocellular Carcinoma	58.0%	[77]	Extrapolated by 1-year survival rate
		Small Cell Lung Cancer	62.9%	[22]	Extrapolated by 1-year survival rate
**Median Time from Diagnosis to Death**	The most recently-available median time from diagnosis to death, reported in months.	Squamous Cell Carcinoma of H&N	Not Found		
		Well-Differentiated Thyroid	Not Found		
		Merkel Cell Carcinoma	Not Found		
		Gastrointestinal Stromal Tumor	Not Found		
		Small Cell Lung Cancer	7	[80]	
		Renal Cell Carcinoma	10	[94]	
		Hepatocellular Carcinoma	11	[95]	Germany
		Pleural Mesothelioma	11.3	[96]	NHS Grampian (Scotland)
		Glioblastoma	12	[68]	Thailand
		Melanoma (Skin)	27.6	[97]	2.3 years
		NSCLC, EGFR+	36.9	[49]	Japan
		Gastric Carcinoma	51	[98]	outside US
		NSCLC, ALK+	55.4	[49]	Japan
		Urothelial Carcinoma	63.2	[99]	Iran
		**Pulmonary Arterial Hypertension**	**72**	**[54]**	**From the literature, it is between 5-7 years.**
		Medullary Thyroid Cancer	103.2	[33]	8.6 years
**Functional Status Scale**	If there is a corresponding functional status scale or holistic staging framework for the analog disease, reported as 1.	Renal Cell Carcinoma	0	[100]	EORTC Quality of Life Questionnaires
		Merkel Cell Carcinoma	0	[100]	EORTC Quality of Life Questionnaires
		**Pulmonary Arterial Hypertension**	**1**	**[14]**	**World Health Organization functional class; 6-minute walk test**
		Gastric Carcinoma	1	[100]	EORTC Quality of Life Questionnaires
		Glioblastoma	1	[100]	EORTC Quality of Life Questionnaires
		Hepatocellular Carcinoma	1	[100]	EORTC Quality of Life Questionnaires
		Medullary Thyroid Cancer	1	[100]	EORTC Quality of Life Questionnaires
		NSCLC, EGFR+	1	[100]	EORTC Quality of Life Questionnaires
		Melanoma (Skin)	1	[100]	EORTC Quality of Life Questionnaires
		NSCLC, ALK+	1	[100]	EORTC Quality of Life Questionnaires

Disease-Level Characteristic	Dimension Raw Values and Data Attributes	Disease Name	Raw Data Value	Reference	Notes
		Pleural Mesothelioma	1	[100]	EORTC Quality of Life Questionnaires
		Small Cell Lung Cancer	1	[100]	EORTC Quality of Life Questionnaires
		Squamous Cell Carcinoma of H&N	1	[100]	EORTC Quality of Life Questionnaires
		Urothelial Carcinoma	1	[100]	EORTC Quality of Life Questionnaires
		Well-Differentiated Thyroid	1	[100]	EORTC Quality of Life Questionnaires
		Gastrointestinal Stromal Tumor	1	[100]	EORTC Quality of Life Questionnaires
**Mobility Problem**	If the mobility problem is a prominent sequela for the disease, reported as 1; otherwise reported as 0.	NSCLC, ALK+	Not Found		
		Pleural Mesothelioma	Not Found		
		Merkel Cell Carcinoma	Not Found		
		Gastric Carcinoma	0	[101]	China, 75.7% of patients with gastric cancer in a hospital in Suzhou, China reported with "no mobility problem".
		Hepatocellular Carcinoma	0	[102]	The mean EQ-5D-5L score for mobility for patients with hepatocellular carcinoma in CELESTIAL trial was 1.39, median was 1.00 (where value of 1 = "no mobility problems" in EQ-5D-5L). Therefore, assuming mobility problems are not a prominent sequalae.
		Medullary Thyroid Cancer	0	[103]	Japan, 64% of medullary thyroid cancer patients report no mobility problems on EQ-5D-5L.
		NSCLC, EGFR+	0	[104]	80% of EGFR+ NSCLC patients reported no mobility problems on the EQ-5D-5L.
		Melanoma (Skin)	0	[105]	Australia; 80.7% of patients reported with no mobility problem
		Squamous Cell Carcinoma of H&N	0	[106]	63.2% of them reported with "no mobility problem". (EQ-5D-5L)
		Urothelial Carcinoma	0	[107]	63% of patients reported "have no mobility problem". (EQ-5D-5L)
		Well-Differentiated Thyroid	0	[103]	67% of patients report no mobility problems on EQ-5D-5L.
		Gastrointestinal Stromal Tumor	0	[108]	56.8% of placebo-treated GIST patients reported no mobility problems on EQ-5D-5L.
		**Pulmonary Arterial Hypertension**	**1**	**[15]**	

Disease-Level Characteristic	Dimension Raw Values and Data Attributes	Disease Name	Raw Data Value	Reference	Notes
		Glioblastoma	1	[109]	48.8% of patients with bilioblastoma report no problems in mobility
		Renal Cell Carcinoma	1	[110]	Metastatic renal cell carcinoma; Netherland; Around 32% of patients reported no mobility problem
		Small Cell Lung Cancer	1	[111]	49% of SCLC patients reported no mobility problems on the EQ-5D-5L.
**Quality-Adjusted Life Years (QALYs)**	The most recently-available estimate of average lifetime QALYs, which quantifies the health effect of a medical intervention or treatment, for the analog disease.	Well-Differentiated Thyroid	Not Found		
		Merkel Cell Carcinoma	Not Found		
		Squamous Cell Carcinoma of H&N	0.82	[112]	The QALYs are averaged across: Nivolumab, Pembrolizumab
		Small Cell Lung Cancer	0.9	[113]	The QALYs are averaged across: Etoposide, Etoposide-Pembrolizumab
		Hepatocellular Carcinoma	1.021	[114]	SOC
		NSCLC, ALK+	1.2	[115]	The QALYs are averaged across: alectinib, ceritinb
		Pleural Mesothelioma	1.32	[116]	The QALYs are averaged across: Nivolumab, Ipilimumab, Chemotherapy
		NSCLC, EGFR+	1.34	[117]	The QALYs are averaged across: CIS-PEM, AFAT, ERLO, GEFI
		Glioblastoma	1.61	[118]	SOC
		Gastric Carcinoma	1.72	[119]	
		Medullary Thyroid Cancer	1.79	[120]	SOC
		Melanoma (Skin)	2.99	[121]	The QALYs are averaged across: til-nki, ipilimumab
		Renal Cell Carcinoma	3.11	[122]	The QALYs are averaged across Sunitinib, Nivolumab-Ipilimumab ,Atezolizumab-Bevacizumab, Avelumab + Axitinib, Nivolumab + cabozantinib, Lenvatinib + Pembrolizumab, Pembrolizumab + Axitinib
		**Pulmonary Arterial Hypertension**	**4.00**	**[39-41]**	**QALYs of Standard Care: 2.95**<br>**Macitentan: 5.655**<br>**Bosentan: 4.76**<br>**Sildenafil: 3.974**<br>**Tadalafil: 3.508**<br>**Supportive Care: 2.952**<br>**Ambrisentan 5mg: 3.907**<br>**Ambrisentan 10mg: 3.63**<br>**Riociguat: 3.645**<br>**Average QALYs including SOC = 4.00**

Disease-Level Characteristic	Dimension Raw Values and Data Attributes	Disease Name	Raw Data Value	Reference	Notes
					**The QALYs are averaged if a product has different QALYs for different populations.**
		Urothelial Carcinoma	4.93	[48]	The QALYs are averaged across: Nadofaragene Firadenovec, Oportuzumab Monatox
		Gastrointestinal Stromal Tumor	7.18	[123]	1-year treatment of imatinib
**Disability-Adjusted Life Years (DALYs)**	The most recently-available average number of DALYs, defined as the number of lost years of "healthy" life, for the analog disease.	Glioblastoma	Not Found		
		Medullary Thyroid Cancer	Not Found		
		NSCLC, EGFR+	Not Found		
		NSCLC, ALK+	Not Found		
		Renal Cell Carcinoma	Not Found		
		Small Cell Lung Cancer	Not Found		
		Squamous Cell Carcinoma of H&N	Not Found		
		Merkel Cell Carcinoma	Not Found		
		Gastrointestinal Stromal Tumor	Not Found		
		**Pulmonary Arterial Hypertension**	**2.56**	**[27,28]**	**Estimated from 640,000 DALYs and 250,412 PAH patients globally**
		Well-Differentiated Thyroid	11.20	[26]	
		Pleural Mesothelioma	11.37	[124]	
		Melanoma (Skin)	60.6	[125]	
		Urothelial Carcinoma	68.10	[126]	
		Gastric Carcinoma	75.7	[127]	
		Hepatocellular Carcinoma	112.6	[128]	

Table S8. Data Collected on Therapeutic Landscape Disease-level Characteristics

Disease-Level Characteristic	Dimension Raw Values and Data Attributes	Disease Name	Raw Data Value	Reference
**Available Unique Products**	The number of unique products (based on active moiety, irrespective of mode of delivery) available on the U.S. market for the analog disease.	Medullary Thyroid Cancer	3	[17]
		Pleural Mesothelioma	3	[17]
		Merkel Cell Carcinoma	3	[17]
		Gastrointestinal Stromal Tumor	4	[17]
		Small Cell Lung Cancer	5	[17]
		Squamous Cell Carcinoma of H&N	5	[17]
		Well-Differentiated Thyroid	5	[17]
		Glioblastoma	7	[17]
		Urothelial Carcinoma	8	[17]
		**Pulmonary Arterial Hypertension**	**10**	**[17]**
		NSCLC, ALK+	10	[17]
		Hepatocellular Carcinoma	12	[17]
		Gastric Carcinoma	13	[17]
		NSCLC, EGFR+	13	[17]
		Melanoma (Skin)	16	[17]
		Renal Cell Carcinoma	16	[17]
**Number of Generic/Biosimilar Products Available**	The number of unique products (based on active moiety, irrespective of mode of delivery) that have at least one generic/biosimilar products available on the U.S. market for the analog disease.	Medullary Thyroid Cancer	0	[17]
		Merkel Cell Carcinoma	0	[17]
		Hepatocellular Carcinoma	1	[17]
		NSCLC, ALK+	1	[17]
		Pleural Mesothelioma	1	[17]
		Urothelial Carcinoma	1	[17]
		Gastrointestinal Stromal Tumor	1	[17]
		Melanoma (Skin)	2	[17]
		Small Cell Lung Cancer	2	[17]
		Squamous Cell Carcinoma of H&N	2	[17]
		Well-Differentiated Thyroid	2	[17]
		Gastric Carcinoma	3	[17]
		Glioblastoma	3	[17]
		NSCLC, EGFR+	4	[17]
		**Pulmonary Arterial Hypertension**	**6**	**[17]**
		Renal Cell Carcinoma	7	[17]
Footnote: [1] Ipilimumab and nivolumab exist as combination therapy				

Table S9. Data Collected on Healthcare Resource Utilization Disease-level Characteristics

Disease-Level Characteristic	Dimension Raw Values and Data Attributes	Disease Name	Raw Data Value	Reference	Notes
**Annual Hospitalization Rate**	The most recently-available annual hospitalization rate for the analog disease, reported as a percentage (%).	Medullary Thyroid Cancer	Not Found		
		NSCLC, ALK+	Not Found		
		Medullary Thyroid Cancer	Not Found		
		NSCLC, ALK+	Not Found		
		Glioblastoma	13.3%	[129]	
		Renal Cell Carcinoma	16.8%	[130]	
		Small Cell Lung Cancer	20.2%	[131]	
		Melanoma (Skin)	22.0%	[132]	Monthly
		NSCLC, EGFR+	26.8%	[53]	
		**Pulmonary Arterial Hypertension**	**38.80%**	**[51]**	
		Gastrointestinal Stromal Tumor	44.0%	[52]	
		Merkel Cell Carcinoma	45.3%	[133]	
		Squamous Cell Carcinoma of H&N	45.4%	[134]	Study population includes patients with recurrent/metastatic SCCHN; the hospitalization in the follow-up of 9.9 month
		Well-Differentiated Thyroid	53.2%	[135]	study population is in differentiated thyroid cancer patients who are refractory to radioactive iodine refractory from US and EU-5 (France, Germany, Italy, Spain, and UK)
		Hepatocellular Carcinoma	67.0%	[136]	
		Gastric Carcinoma	68.90%	[134]	
		Pleural Mesothelioma	85.4%	[137]	
		Urothelial Carcinoma	97.2%	[138]	

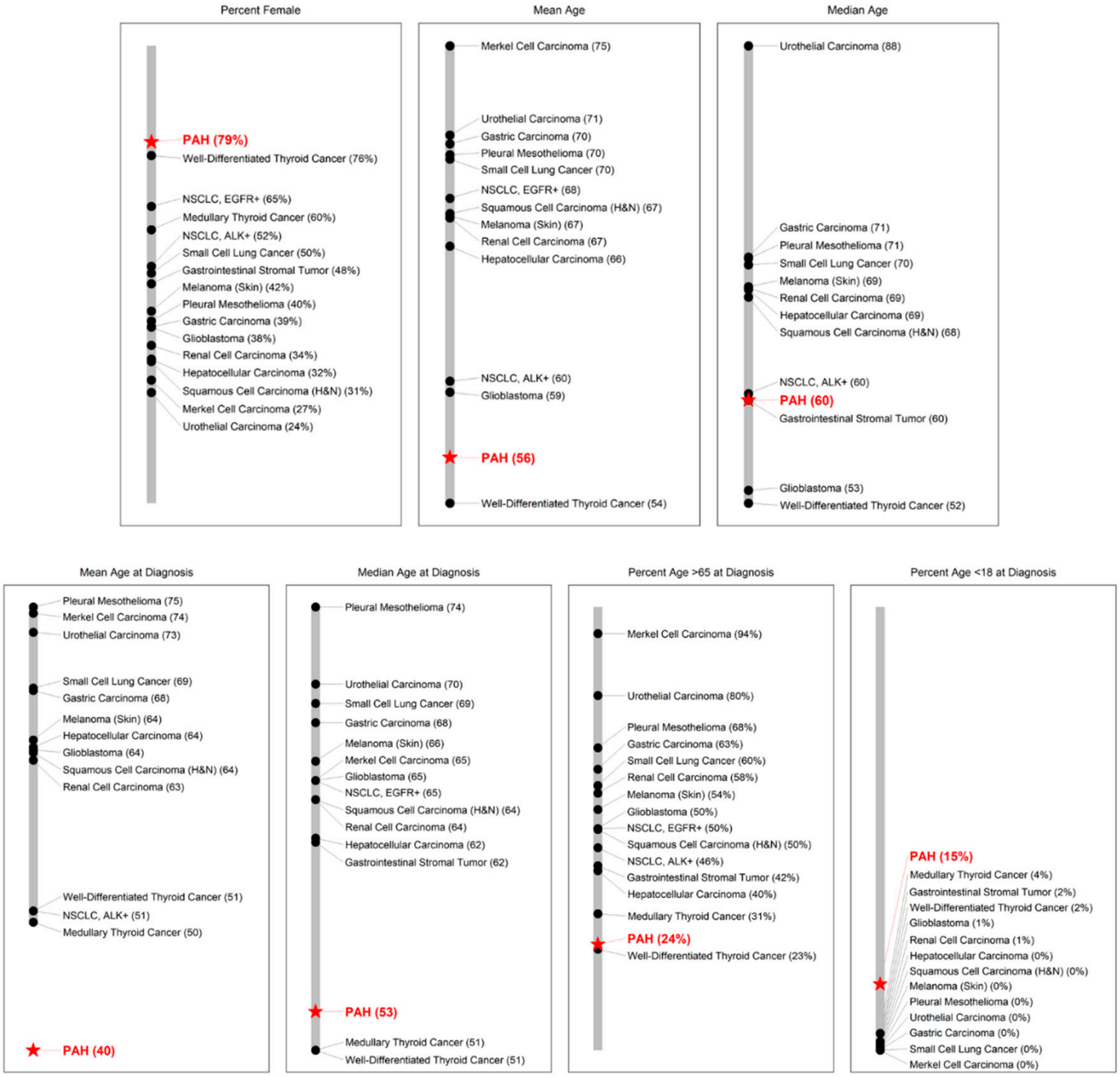

Figure S1. Distribution of Cancer Analogs Within Each Epidemiology Disease-level Metric

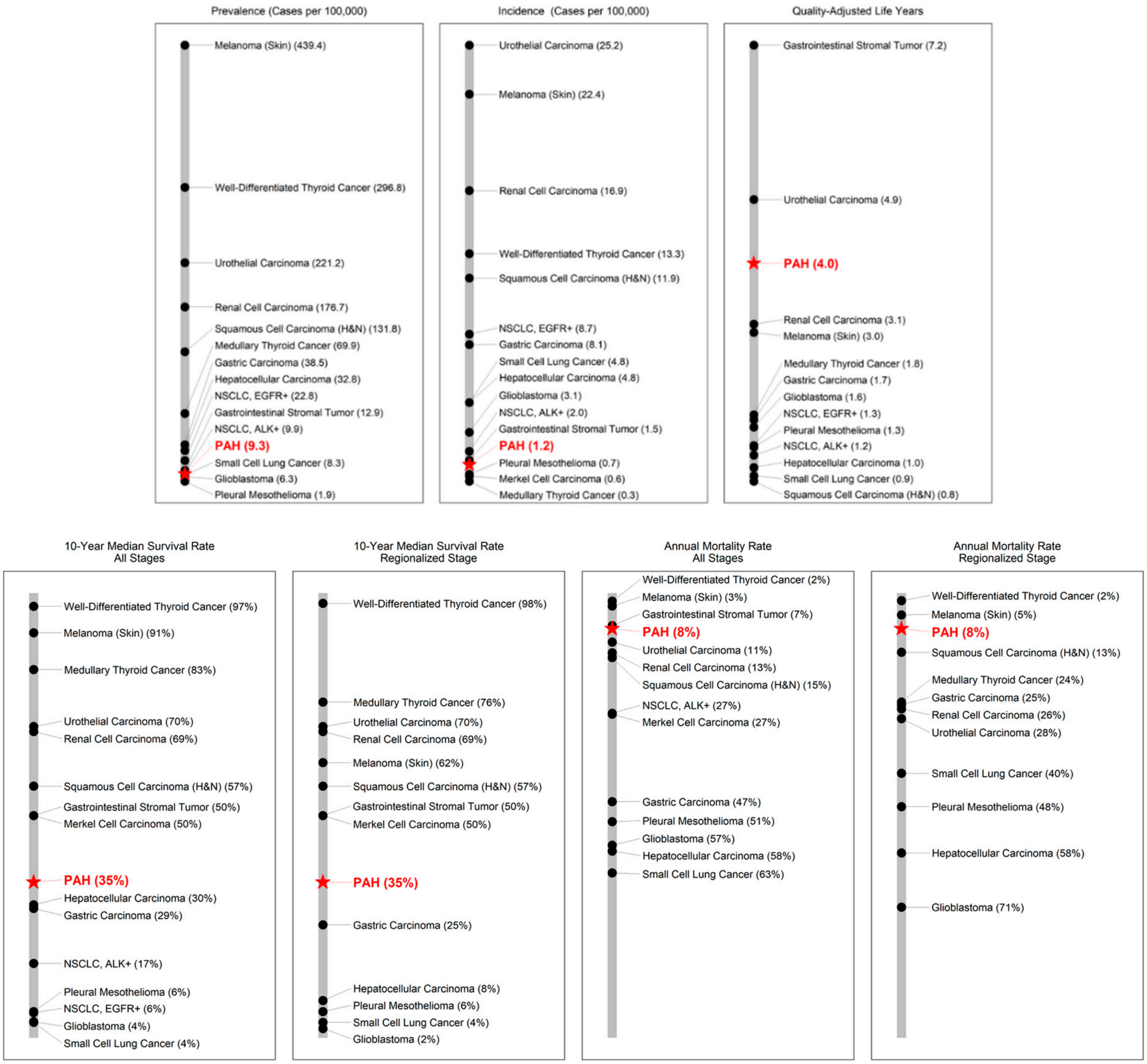

Figure S2. Distribution of Cancer Analogs Within Each Clinical Landscape Disease-level Metric

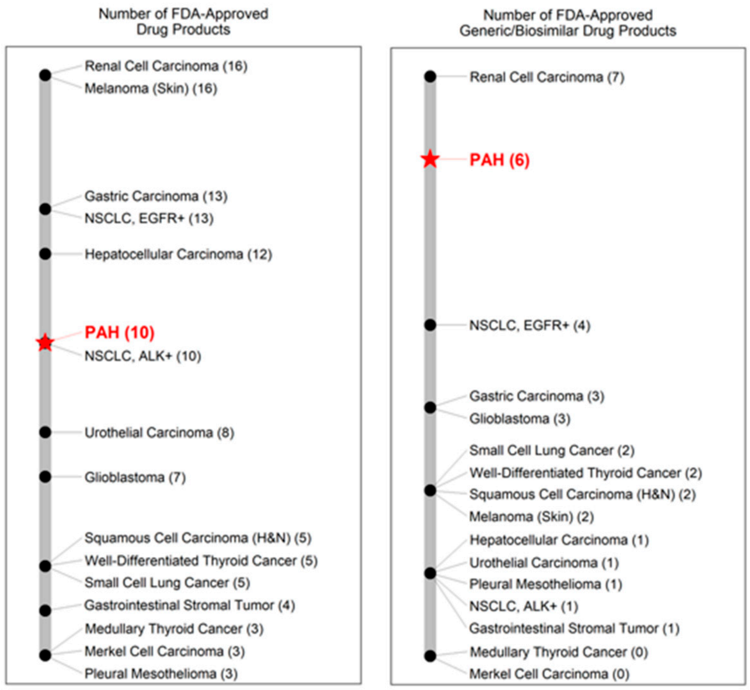

Figure S3. Distribution of Cancer Analogs Within Each Treatment Landscape Disease-level Metric

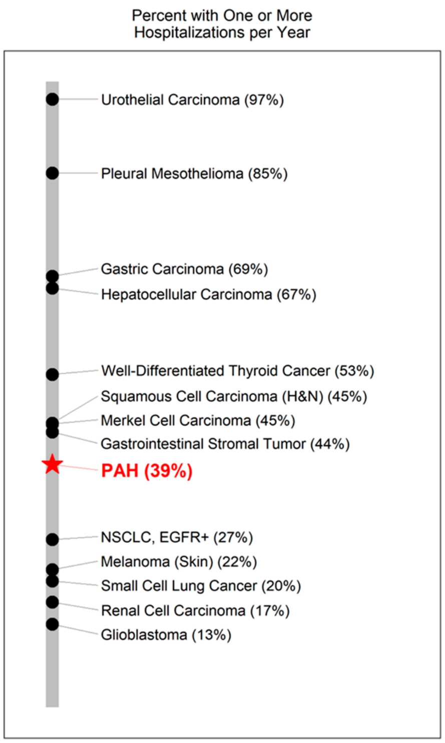

Figure S4. Distribution of Cancer Analogs Within Each Healthcare Utilization Landscape Disease-level Metric
